# Correlation between immune-related adverse events and efficacy of PD-(L)1 inhibitors in small cell lung cancer: a multi-center retrospective study

**DOI:** 10.1186/s12931-024-02890-3

**Published:** 2024-06-21

**Authors:** Jian Zhang, Aiqin Gao, Shuyun Wang, Yanxin Sun, Jiake Wu, Dahai Wang, Yihui Ge, Juan Li, Haifeng Sun, Qinglei Cheng, Yuping Sun

**Affiliations:** 1 Phase I Clinical Research Center, Shandong Cancer Hospital and Institute, Shandong First Medical University, Shandong Academy of Medical Sciences, 440 Jiyan Road, Jinan, 250117 Shandong China; 2 Department of Thoracic Radiation Oncology, Shandong Cancer Hospital and Institute, Shandong First Medical University, Shandong Academy of Medical Sciences, Jinan, 250117 Shandong China; 3 School of Clinical Medicine, Weifang Medical University, Weifang, 250117 Shandong China; 4 Phase I Clinical Research Center, Shandong University Cancer Center, Jinan, 250117 Shandong China

**Keywords:** Extensive-stage small cell lung cancer (ES-SCLC), Immunotherapy, Programmed cell death protein 1 (PD-1), Programmed cell death ligand protein 1 (PD-L1), Immune-related adverse events (irAEs)

## Abstract

**Background:**

Patients receiving PD-(L)1 inhibitors frequently encounter unusual side effects known as immune-related adverse events (irAEs). However, the correlation of irAEs development with clinical response in small cell lung cancer (SCLC) is unknown.

**Method:**

This retrospective study enrolled 244 stage IV SCLC patients who receiving PD-(L)1 inhibitors from 3 cancer centers. The correlation of irAEs with objective response rate (ORR), disease control rate (DCR), progression-free survival (PFS), and overall survival (OS) were evaluated.

**Results:**

140 in 244 (57%) patients experienced irAEs, with 122 (87.1%) experiencing one and 18 (12.9%) experiencing two or more. Compared to patient without irAEs, those developing irAEs had higher ORR (73.6% vs. 52.9%, *P* < 0.001) and DCR (97.9% vs. 79.8%, *P* < 0.001), as well as prolonged median PFS (8.8 vs. 4.5 months, *P* < 0.001) and OS (23.2 vs. 21.6 months, *P* < 0.05). Among the different spectra of irAEs, thyroid dysfunction, rash, and pneumonitis were the most powerful indicator for improved PFS. When analyzed as a time-dependent covariate, the occurrence of irAEs was associated with significant improvement in PFS rather than in OS. Furthermore, patients experiencing multisystem irAEs displayed a longer PFS and OS compared with single-system irAEs and the irAE-free ones. IrAEs grade and steroid use did not impact the predictive value of irAEs on PFS.

**Conclusion:**

The presence of irAEs predicts superior clinical benefit in SCLC. Patients who develop multi-system irAEs may have an improved survival than those developed single-system irAEs and no-irAEs. This association persists even when systemic corticosteroids were used for irAEs management.

**Supplementary Information:**

The online version contains supplementary material available at 10.1186/s12931-024-02890-3.

## Introduction

Lung cancer is the leading cause of cancer deaths worldwide, with an estimated 1.8 million deaths (18%) in 2020 [1]. SCLC accounts for about 15% of all lung cancers and is distinguished by high proliferation rate, intense early metastasis at diagnosis, and poor prognosis [2]. Although chemotherapy using platinum and etoposide have been the standard regimens for SCLC for approximately 20–30 years, recent efforts to target the immune checkpoint inhibitor PD-(L)1 have changed the treatment paradigm in SCLC. The PD-L1 inhibitors atezolizumab and durvalumab, in combination with platinum and etoposide, have significantly prolonged overall survival (OS) and reduced the risk of death in SCLC patients in IMpower 133 and CASPIAN studies, and are approved as standard first-line treatment of extensive-stage SCLC in 2019 and 2020, respectively [3, 4]. Afterwards, adebrelimab, another PD-L1 inhibitor, displayed similar survival benefit in CAPSTONE-1 study and was approved as first-line options in 2023 by National Medical Products Administration (NMPA) of China [5]. Serplulimab is the only PD-1 inhibitor approved in extensive-stage small-cell lung cancer(ES-SCLC), based on the remarkable extension of PFS and OS in ASTRUM 005 study [6].

Immunotherapy improves efficacy but is associated with adverse events that differ from those seen with conventional therapy. The immune response attacks not only tumors but also normal tissues, resulting in irAEs in cutaneous, gastrointestinal, hepatic, endocrine and other systems [7]. In non-small cell lung cancer (NSCLC), melanoma, and renal cell carcinoma, the occurrance of irAEs is identified as a crucial predictor for better efficacy of immunotherapy [8–10]. However, whether such an association exists in SCLC has not been described. Here, by retrospectively analyzing the real-world data, we investigated a link between development of irAEs and clinical outcomes in SCLC patients treated with PD-(L)1 inhibitors.

## Methods

### Patient selection

ES-SCLC patients treated with PD-(L)1 inhibitors monotherapy or combined with chemotherapy were retrospectively analyzed from Shandong Cancer Hospital, Shandong Provincial Hospital, and Qilu Hospital of Shandong University between January 2018 and June 2022 (eFigure 1 in the Supplement). Patients who were initially diagnosed as LS-SCLC but later experiencing recurrence or progression, and re-staged as ES-SCLC were included. First-line anti-PD-(L)1 immunotherapy for limited-stage SCLC, or concurrently with other cancer types were excluded (eFigure 1 in the Supplement). IrAEs were defined as adverse events with a potential immunologic basis that required close monitoring and/or potential intervention with immunosuppressives or hormone replacement. Thyroid function was evaluated at baseline and every 6 weeks thereafter. Patient symptoms and physical exploration and laboratory data were assessed at every cycle. Patients were followed up every 3 month during and after ICIs until occurrence of death or lost follow-up. Endocrine system toxicity, skin reactions, immune pneumonia, gastrointestinal reactions, cardiovascular system reactions, and neurological reactions are examples of irAEs. Multisystem irAEs were defined as irAEs involving more than one organ or system. Based on the occurrence of irAEs, patients were divided into irAEs versus no-irAEs groups.

### Data collection and irAE assessment

Basic clinical characteristics, PD-1 therapy types and dosages, irAEs types, onset, severity, management, and prognosis of patients are all collected. The National Cancer Institute Common Adverse Event Terminology Criteria Version 5.0 was used to evaluate grading and classification criteria. SCLC was staged by combining the VALG staging method with the TNM staging system [11]. Radiological assessments were performed every 6–8 weeks to determine the best objective efficacy of treatment using the Response Evaluation Criteria in Solid Tumours (RECIST) (version 1.1). The objective remission rate (ORR), disease control rate (DCR), progression-free survival (PFS), and OS were used to assess the efficacy of PD-(L)1 inhibitors. ORR was defined as the ratio of patients who obtain partial or complete remission, while DCR as the ratio of patients with partial, complete, or stable disease. Progression-free survival is defined as the time from the start of ICIs to progression or death (PFS) from any cause. The time from the start of ICIs to death or the most recent visit was defined as OS.

### Statistical analysis

Categorical and continuous variables were descriptively summarized using percentages and medians. Patients’ baseline clinical characteristics in both groups were examined. The Mann-Whitney U rank sum test was used to estimate age and number of immunotherapy cycles, the Pearson χ2 test was used to test dichotomous variables such as gender, smoking status, and ECOG PS, and the R × C table χ2 test was used to test history of radiation therapy. The Kaplan-Meier method was used to estimate the PFS and OS analyses. A two-sided log-rank test was used to estimate the risk of irAE. To compare the time to the first irAE in multiple groups of irAE patients, the Kruskal-Wallis rank sum test was used. To compare the time to the first irAE occurrence in patients with single versus multiple system irAEs, the Mann-Whitney U rank sum test was used. Univariate and multivariate COX regression risk proportional model analysis were used to assess differences in PFS and OS between irAEs groups, with *P* < 0.05 considered a statistically significant difference. SPSS 26.0 was used for all statistical analyses. We treated irAE as a time-dependent covariate to avoid lead-time bias caused by its time-dependent nature [12].

## Results

### Patient characteristics

244 SCLC patients treated with ICIs were included from 3 cancer centers. Table 1 showed the baseline characteristics of patients based on irAEs development. The majority of patients (*N* = 210, 86.1%) were at the extensive stage when they were diagnosed. PD-L1 inhibitors were more frequently used (*N* = 152, 62.3%) than PD-1 inhibitors (*N* = 92, 37.7%). The baseline clinical characteristics of the irAEs and no-irAEs groups were largely balanced. We observed slightly higher proportion of patients receiving first-line immunotherapy in irAEs group (80% vs. 69.2%, *P* = 0.053) compared with no-irAEs group. In addition, median ICI duration was longer in the irAEs group than in the no-irAEs group (8.0 months vs. 5.0 months, *P* < 0.001).

**Table 1:** Baseline characteristics of SCLC patients with Versus without irAEs

Variables	All patients (*N* = 244)	No irAEs (*N* = 104)	irAEs (*N* = 140)	*P* value^a^
Age (y), median (range)	62(57–68)	61(56–68)	63(57–68)	0.575
Gender(%male)	198(81.1%)	86(82.7%)	112(80.0%)	0.595
Smoking (%)	163(66.8%)	71(68.3%)	92(65.7%)	0.675
Stage at diagnosis (%Extensive)	210(86.1%)	87(83.7%)	123(87.9%)	0.348
ECOG PS(%)				0.659
0 ∼ 1	118(48.4%)	52(50.0%)	66(47.1%)	
≥ 2	126(51.6%)	52(50.0%)	74(52.9%)	
Line of therapy for ICIs(%)				0.053
1st	184(75.4%)	72(69.2%)	112(80.0%)	
≥ 2nd	60(24.6%)	32(30.8%)	28(20.0%)	
Any history of brain metastases before ICI (%)	76(31.1%)	28(26.9%)	48(34.3%)	0.219
Any history of liver metastases before ICI (%)	73(29.9%)	33(31.7%)	40(28.6%)	0.594
Treatment received(%)				0.312
PD-1	92(37.7%)	43(41.3%)	49(35.0%)	
PD-L1	152(62.3%)	61(58.7%)	91(65.0%)	
Treatment regimens(%)				0.418
anti PD-(L)1 monotherapy	4(1.6%)	3(2.9%)	1(0.7%)	
anti PD-(L)1 + chemotherapy	240(98.4%)	101(97.1%)	139(99.3%)	
ICI treatment cycles	6.0(5.0–8.0)	5.0(3.0–7.0)	8.0(6.0–11.0)	**<0.001**

Abbreviations: ECOG PS, Eastern Cooperative Oncology Group performance status; ICI, immune checkpoint inhibitor; irAE, immune-related adverse event; PD-L1, programmed death-ligand 1; PD-1, programmed cell death protein 1

^a^ Categorical and continuous variables were compared using χ2 and Kruskal-Wallis tests, respectively

### Immune-related adverse events

#### Spectrum of irAEs

eTable 1 showed the spectrum of irAEs in our patient cohort. 140 (57%) of the 244 patients who received immunotherapy developed irAEs, with 122 (87.1%) experiencing one and 18 (12.9%) experiencing two or more. Only 6 patients (3.6%) had grade 3 to 4 irAEs, including rash (*N* = 2), pneumonitis (*N* = 1), hyperthyroidism (*N* = 1), liver disease (*N* = 1), and type 1 diabetes mellitus (*N* = 1). 3 patients were permanently discontinued as a result of irAEs, but no patients died as a result of irAEs. Endocrine system toxicity (*N* = 131, 79.4%), skin reactions (*N* = 16, 9.7%), and immune pneumonia (*N* = 10, 6.1%) were the most common irAEs (eTable 1 in the Supplement). Hypothyroidism combined with rash (*N* = 6, 33.3%), hypothyroidism combined with pneumonia (*N* = 4, 22.2%), and pneumonitis combined with rash (*N* = 3, 16.7%) were the most common multisystem irAEs (eFigure 2 A in the Supplement). 15 of the 29 patients were treated for irAEs with corticosteroids, including 8 cases of immune pneumonia.

#### Time to Onset of irAEs

The time to first irAE in all patients was 2.5 months (Range:1.4–4.4). The time to first irAE varied among different irAEs, with the earliest irAE occurring as rash (1.8 months) (eFigure 2 B in the Supplement). No statistically difference was found in the time to first irAE in patients with single-system versus multi-system irAEs (2.4 vs. 2.6 months, *P* = 0.48) (eFigure 2 C in the Supplement).

#### Risk Factors for irAEs

Longer ICI duration was found to be an independent risk factor for the occurrence of irAEs in both univariate (OR: 1.30; 95% CI: 1.18–1.44; *P* < 0.001) and multivariate (OR: 1.32; 95% CI: 1.19–1.46; *P* < 0.001) log-rank test analyses (eTable 2 in the Supplement).

### Correlation of irAEs with treatment response and outcomes

#### IrAEs group vs. no-irAEs group

**Fig. 1:**
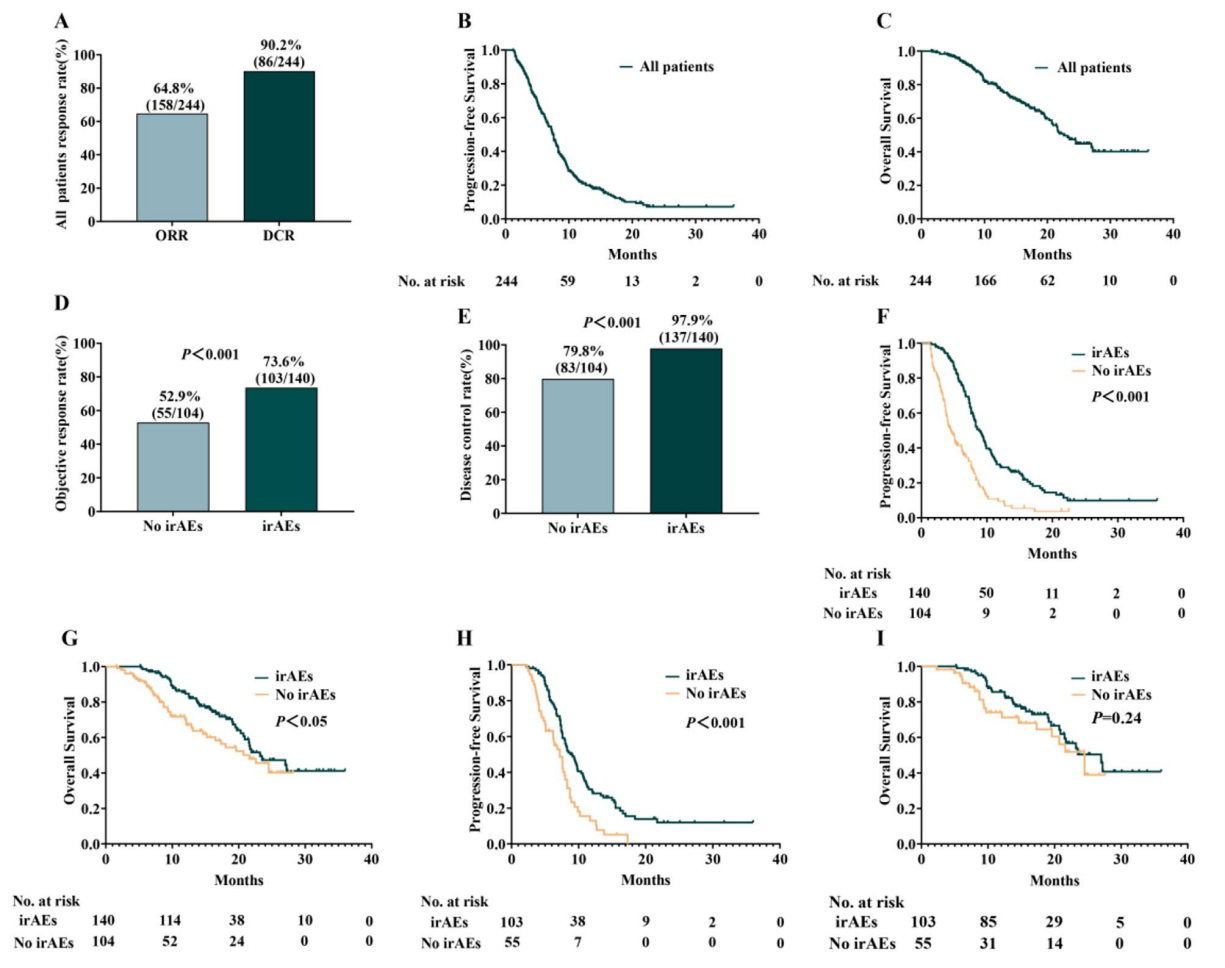
(**A**) The proportion of patients who achieved response and disease control to immunotherapy in all patients. (**B** and **C**) PFS (**B**) and OS (**C**) in all SCLC patients treated with immunotherapy. (**D** and **E**) The ORR (**D**) and DCR (**E**) in the irAEs group and no-irAEs group. (**F** and **G**) PFS (**F**) and OS (**G**) in patients with/without irAEs. (**H** and **I**) PFS (**H**) and OS (**I**) of respondents with/without irAEs

The median follow-up was 22.2 months (95% CI: 19.6–24.8) at the time of data analysis, with an ORR of 64.8% and a DCR of 90.2% in the study population (Fig. 1A). The median PFS (Fig. 1B) and OS (Fig. 1C) were 7.5 months (95% CI, 6.8–8.2) and 22.6 months (95% CI, 19.8–25.4), respectively. The ORR (Fig. 1D) and DCR (Fig. 1E) were higher in the irAEs group than in the no-irAEs group (ORR: 73.6% vs. 52.9%, *P* < 0.001; DCR: 97.9% vs. 79.8%, *P* < 0.001). The irAEs group outlived the no-irAEs group in terms of median PFS (8.8 months vs. 4.5 months, *P* < 0.001, Fig. 1F) and median OS (23.2 months vs. 21.6 months, *P* < 0.05, Fig. 1G). Even in responders, we found significantly prolonged PFS in the irAEs group compared with the no-irAEs group (8.8 months vs. 7.2 months, *P* < 0.001, Fig. 1H). However, we did not observed OS extension of the irAEs group in the responders (27.0 months vs. 24.5 months, *P* = 0.24, Fig. 1I).

To explain the time-dependent nature of irAEs, the development of irAEs was treated as a time-varying covariate in univariate and multivariate Cox proportional hazard models (eTable 3 in the Supplement). Univariate analysis revealed that the occurrence of irAEs (HR: 0.94; 95% CI: 0.91–0.97; *P* < 0.001), being female (HR:0.68; 95% CI:0.47–0.98; *P* < 0.05), no smoking (HR:0.72; 95% CI:0.53–0.96; *P* < 0.05), first-line ICI usage (HR:0.65; 95% CI:0.47–0.89; *P* < 0.01), and no history of liver metastases before ICI (HR:0.74; 95% CI:0.55–0.99; *P* < 0.05) were all associated with longer PFS (eTable 3 in the Supplement). In multifactorial analysis, the occurrence of irAEs (HR:0.93; 95% CI:0.90–0.97; *P* < 0.001), first-line ICI usage (HR:0.57; 95% CI:0.35–0.93; *P* < 0.05), and without liver metastases before ICI (HR:0.73; 95% CI:0.54–0.99; *P* < 0.05) predicted a longer PFS (eTable 3 in the Supplement). In addition, univariate analysis (HR:0.63; 95% CI:0.41–0.96; *P* < 0.05) and multivariate analysis (HR:0.59; 95% CI:0.38–0.93; *P* < 0.05) revealed that treatment with PD-L1 inhibitors had a longer OS compared with PD-1 inhibitors (eTable 3 in the Supplement). Neither univariate nor multivariate analysis showed a link between OS and the prevalence of irAEs (eTable 3 in the Supplement). When analyzed by different irAE spectrum, we found that thyroid dysfunction (HR:0.37; 95% CI:0.27–0.50; *P* < 0.001), rash (HR:0.27; 95% CI:0.14–0.51; *P* < 0.001), and immunological pneumonia (HR:0.36; 95% CI:0.18–0.72; *P* < 0.01) were linked to better PFS in the patient cohort (eTable 4 in the Supplement). However, only thyroid dysfunction was correlated with better OS (HR:0.62; 95% CI:0.40–0.96; *P* < 0.05) in univariate rather than in multivariate analysis (eTable 4 in the Supplement).

#### Multi-system irAEs group vs.single-system irAEs group vs.no-irAEs group

**Fig. 2:**
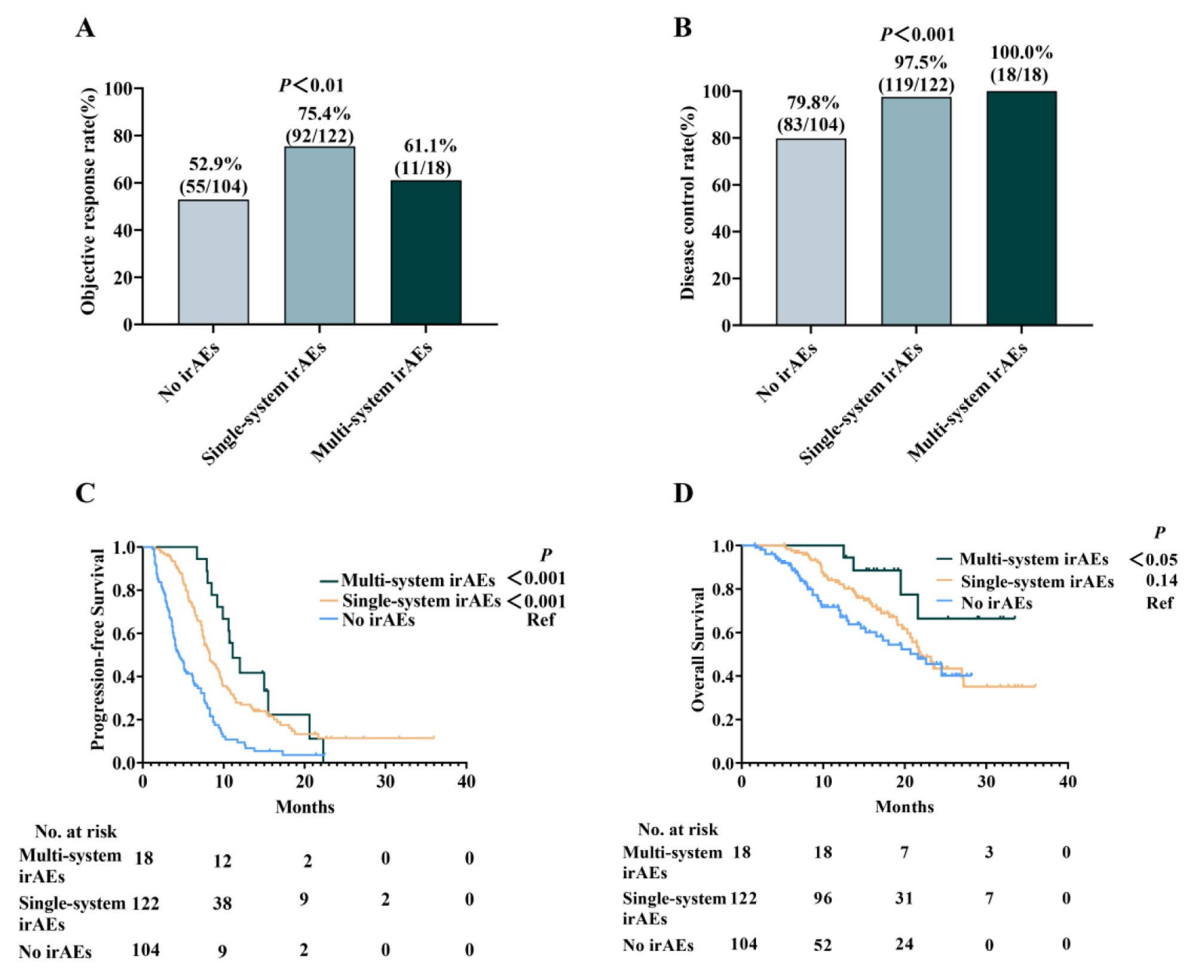
(**A** and **B**) The ORR (**A**) and DCR (**B**) in patients with multi-system irAEs, single-system irAEs and without irAEs. (**C** and **D**) PFS (**C**) and OS (**D**) in patients with multi-system irAEs, single-system irAEs and without irAEs

Since there is no data regarding multisystem irAEs in SCLC despite the fact that they are associated with improved survival in NSCLC [12, 13], we further explored patient outcomes based on multi-system and single-system irAE development. The ORR were 61.1%, 75.4% and 52.9% (*P* < 0.01) in the multi-system irAEs, single-system irAEs, and no-irAEs groups, respectively (Fig. 2A). While the DCR were 100.0%, 97.5% and 79.8% (*P* < 0.01) in the above groups, respectively (Fig. 2B). Both the multi-system (11.1 months vs. 4.5 months, HR:0.29, 95% CI:0.17–0.50, *P* < 0.001) and single-system irAEs (8.3 months vs. 4.5 months, HR:0.41, 95% CI:0.30–0.56, *P* < 0.001) groups had longer median PFS, but only the multi-system group had a longer median OS (NA vs. 21.6 months, HR:0.39; 95% CI:0.15–0.99; *P* < 0.05) compared to the no-irAEs group (Fig. 2C and D and eTable 5 in the Supplement). Furthermore, there was a significant positive association between irAEs number and PFS (HR:0.46; 95% CI: 0.36–0.59; *P* < 0.001) and OS (HR:0.67; 95% CI:0.47–0.96; *P* < 0.05), respectively (eTable 6 in the Supplement).

#### The correlation of toxicity classification and efficacy

**Fig. 3:**
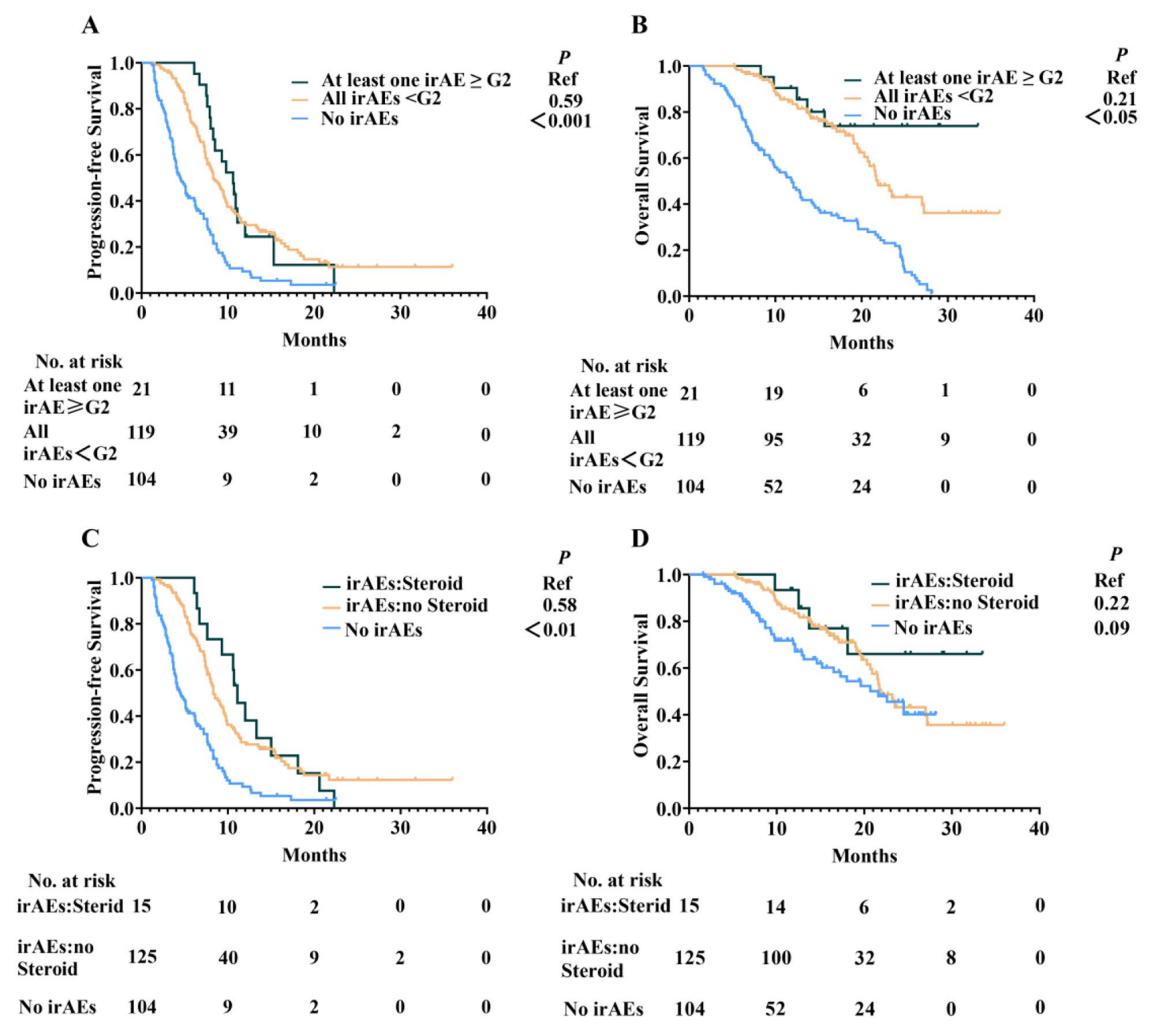
(**A** and **B**) PFS (**A**) and OS (**B**) based on different grade of irAEs. (**C** and **D**) PFS (**C**) and OS (**D**) in steroid treatment group, no steroid group and no-irAEs group

To further analyse the impact of the irAE degree on efficacy, we grouped all irAEs < G2 with at least one irAE ≥ G2. Both groups had longer median PFS (Fig. 3A) and OS (Fig. 3B) compared to the no-irAEs group (10.6 months vs. 4.5 months, *P* < 0.001; NA vs. 21.6 months, *P* < 0.05). However, no difference in PFS and OS was observed between at least one irAE ≥ G2 group and all irAEs < G2 group (Fig. 3A and B). eTable 7 further showed the the prognosis of the Grade 3 or higher irAE cases. Since corticosteroids are the primary treatment agents for irAEs and displayed an immune-suppressive effect, we divided the patients experiencing irAEs into no Steroid group and Steroid group to analyze whether systemic application of corticosteroid had an adverse effect on the clinical outcome of immunotherapy. We found that the Steroid groups had longer median PFS (Fig. 3C) compared to the no-irAEs group (11.1 months vs. 4.5 months, *P* < 0.01), but no difference in OS (Fig. 3D) was observed (NA vs. 21.6 months, *P* = 0.09). However, there is no difference in PFS and OS between Steroid group and no Steroid group (Fig. 3C and D).

## Discussion

Although irAEs were reported to be associated with better outcomes in patients undergoing immunotherapy for melanoma, renal cell carcinoma, and non-small cell lung cancer, their correlation with SCLC is still unknown. In this retrospective multi-center study, we found that patients developing irAEs have superior ORR and DCR, and longer PFS and OS when treated with PD-(L)1 inhibitors. In addition, patients who develop multi-system irAEs displayed improved survival than those who develop single-system irAEs. To our knowledge, this is the first study assessing the correlation of irAEs with the efficacy of PD-(L)1 inhibitors in SCLC.

With the approval of four PD-(L)1 inhibitors as first-line regimens for extensive-stage SCLC, the management of SCLC enters an era of immunotherapy [3–5, 14]. However, precision immunotherapy guided by effective predictive biomarkers for response is still in its infant. Several studies have explored the role of PD-L1 expression or tumor mutational burden (TMB), two well-accepted biomarkers in NSCLC, on predicting immunotherapy response in SCLC, however, the result is still controversial [15–17]. Moreover, a series of potential biomarkers for immunotherapy efficacy are under investigation [18]. Here by retrospectively analyzing the real world data, we identified irAEs as a powerful positive indicator for immunotherapy response. Even after adjusting the ICI duration and restricting the responders, the positive correlation persists. Although the underlying mechanisms are still undetermined, irAEs caused by the activation of autoantigen-specific T cells upon PD-(L)1 inhibitors might indirectly reflect the killing ability of tumor-specific T cells [13]. Similarly, multisystem or high grade of irAEs represent more active systemic immune induced by ICIs, which explains our observation that patients with multisystem or high grade of irAEs seem to have increased PFS and OS compared with their counterparts.

Baseline use of large dose corticosteroids is considered as a negative predictor for immunotherapy benefit in a prevalence of studies [19–22]. However, whether steroid administration for the purpose of irAEs treatment impacts the outcome of SCLC patients is unknown. We found that the use of corticosteroids prolonged PFS and OS in SCLC patients receiving PD-(L)1 inhibitors, compared with the no-irAEs group. We also observed a numerical extension of PFS in Steroid group compared with the no Steroid group. This finding is consistent with the previous reports, which demonstrate that management of irAEs using steroids did not affect the response rate and patient survival in melanoma, NSCLC and renal cell carcinoma [8, 23–25]. These results suggest that systemic corticosteroids should be reasonably used in the setting of irAEs in SCLC.

### Limitations

This study is limited by its retrospective design, which may introduce information bias. In addition, the follow-up period was not sufficiently long to fully evaluate long-term survival outcomes. Furthermore, the small sample size restricted our ability to assess the correlation between various types of irAEs and prognosis. Thus, larger clinical studies or data are necessary for future research. Meanwhile, the diversity of chemotherapeutic agents administered as second-line treatments could account for the observed disparity in PFS between first-line and second-line (or beyond) patients.

### Electronic supplementary material

Below is the link to the electronic supplementary material.

Supplementary Material 1

## Data Availability

No datasets were generated or analysed during the current study.

## Abbreviations

irAEs: Immune-related adverse events
ES-SCLC: Extensive-stage small-cell lung cancer
ORR: Objective response rate
DCR: Disease control rate
PFS: Progression-free survival
OS: Overall survival
TMB: Tumor mutational burden
