# Supplementary material for: Correlation between immune-related adverse events and efficacy of PD-(L)1 inhibitors in small cell lung cancer: a multi-center retrospective study
Source: Respir Res. 2024 Jun 21;25:256. doi: 10.1186/s12931-024-02890-3 (PMC11193240; doi:10.1186/s12931-024-02890-3)
Supplement: Supplementary file 1 — Supplementary Material 1 [file 12931_2024_2890_MOESM1_ESM.docx]

**Supplemental Online Content**

**Jian Zhang, Yuping Sun, Aiqin Gao，et al. Correlation between Immune-Related Adverse Events and Efficacy of PD-1/PD-L1 Inhibitors in Small Cell Lung Cancer: A Multi-center Retrospective Study.**

**eMethods**

**eReferences**

**eTable 1** Summary of immune-related adverse events and their treatment in SCLC treated with immunotherapy.

**eTable 2** Multivariate logistic regression of risk factors for irAEs in SCLC patients treated with immunotherapy.

**eTable 3** Multivariate Cox regression model of overall and progression-free survival in SCLC patients treated with anti-PD(L)1 immunotherapy, utilizing irAEs as a time-varying covariate.

**eTable 4** Multivariate Cox regression model of overall and progression-free survival in SCLC patients treated with anti-PD(L)1 immunotherapy, utilizing the three most common single irAEs as categorical variables.

**eTable 5** Multivariate Cox regression model of overall and progression-free survival in SCLC patients treated with anti-PD(L)1 immunotherapy, utilizing irAEs as a categorical variable.

**eTable 6** Multivariate Cox regression model of overall and progression-free survival in SCLC patients treated with anti-PD(L)1 immunotherapy, utilizing multi-system irAEs as a continuous variable.

**eTable 7** Prognosis of patients with ≥ Grade 3 irAEs.

**eFigure 1.** Study design: inclusion and exclusion criteria.

**eFigure 2.** (A) Chord diagram displaying the spectrum of multi-system irAEs in SCLC patients treated with PD-1/PD-L1 inhibitors. (B and C) Time to onset of multigroup irAEs(B) or multi-system irAEs(C) in SCLC patients treated with PD-1/PD-L1 inhibitors.

This supplemental material has been provided by the authors to give readers additional

information about their work.

**eMethods**

**Study Population**

ES-SCLC patients treated with PD-1/PD-L1 inhibitors combined with etoposide and platinum were retrospectively analyzed from Shandong Cancer Hospital, Shandong Provincial Hospital, and Qilu Hospital of Shandong University between January 2018 and June 2022. Patients with limited-stage SCLC, or concurrently with other cancer types were excluded . IrAEs were defined as adverse events with a potential immunologic basis that required close monitoring and/or potential intervention with immunosuppressives or hormone replacement. Thyroid function was evaluated at baseline and every 6 weeks thereafter. Patient symptoms and physical exploration and laboratory data were assessed at every cycle. Endocrine system toxicity, skin reactions, immune pneumonia, gastrointestinal reactions, cardiovascular system reactions, and neurological reactions are examples of irAE. Multisystem irAEs were defined as irAEs involving more than one organ or system. Based on the occurrence of irAEs, patients were divided into irAEs versus no-irAEs groups.

***Data Collection and irAE Assessment***

Basic clinical characteristics, PD-1 therapy types and dosages, irAE types, onset, severity, management, and prognosis of patients are all collected. The National Cancer Institute Common Adverse Event Terminology Criteria Version 5.0 were used to evaluate grading and classification criteria. SCLC was staged by combining the VALG staging method with the TNM staging system[1]. Radiological assessments were performed every 6-8 weeks to determine the best objective efficacy of treatment using the Response Evaluation Criteria in Solid Tumours (RECIST) (version 1.1). The objective remission rate (ORR), disease control rate (DCR), progression-free survival (PFS), and OS were used to assess the efficacy of PD-1/PD-L1 inhibitors. ORR was defined as the ratio of patients who obtain partial or complete remission, while DCR as the ratio of patients with partial, complete, or stable disease. Progression-free survival is defined as the time from the start of ICI to progression or death (PFS) from any cause. The time from the start of ICI to death or the most recent visit was defined as OS.

***Statistical Analysis***

Categorical and continuous variables were descriptively summarized using percentages and medians. Patients' baseline clinical characteristics in both groups were examined. The Mann-Whitney U rank sum test was used to estimate age and number of immunotherapy cycles, the Pearson χ2 test was used to test dichotomous variables such as gender, smoking status, and ECOG PS, and the R × C table χ2 test was used to test history of radiation therapy. The Kaplan-Meier method was used to estimate the PFS and OS analyses. A two-sided log-rank test was used to estimate the risk of irAE. To compare the time to the first irAE in multiple groups of irAE patients, the Kruskal-Wallis rank sum test was used. To compare the time to the first irAE occurrence in patients with single versus multiple system irAEs, the Mann-Whitney U rank sum test was used. Univariate and multivariate COX regression risk proportional model analysis were used to assess differences in PFS and OS between irAEs groups, with *P* < 0.05 considered a statistically significant difference. SPSS 26.0 was used for all statistical analyses. We treated irAE as a time-dependent covariate to avoid lead-time bias caused by its time-dependent nature[2].

**eReferences**

1. Kalemkerian GP, Gadgeel SM: **Modern staging of small cell lung cancer**. *Journal of the National Comprehensive Cancer Network : JNCCN* 2013, **11**(1):99-104.

2. Shankar B, Zhang J, Naqash AR, Forde PM, Feliciano JL, Marrone KA, Ettinger DS, Hann CL, Brahmer JR, Ricciuti B *et al*: **Multisystem Immune-Related Adverse Events Associated With Immune Checkpoint Inhibitors for Treatment of Non-Small Cell Lung Cancer**. *JAMA oncology* 2020, **6**(12):1952-1956.

**eTable 1 Summary of immune-related adverse events and their treatment in SCLC treated with immunotherapy.**

| **Organ system** | **Any grade**  **(N=165)** | **Specific irAE** | **Grade 1-2**  **(N=159)** | **Grade 3-4**  **(N=6)** | **Therapy**  **(N=29)** |
| --- | --- | --- | --- | --- | --- |
| Endocrine | 36  91  2  1  1 | Hyperthyroidism  Hypothyroidism  Panhypopituitarism  Type 1 diabetes  Hypophysitis | 35  91  2  -  1 | 1  -  -  1  - | 3  5  -  1  - |
| Cutaneous | 16 | Rash | 14 | 2 | 8 |
| Respiratory | 10 | Pneumonitis | 9 | 1 | 8 |
| Gastrointestinal | 1  1  2 | Pancreatitis  Hepatitis  Diarrhea/Colitis | 1  0  2 | -  1  - | -  1  1 |
| Cardiovascular | 2  1 | Myocarditis  Phlebits | 2  1 | -  - | 1  1 |
| Neurologic | 1 | Peripheral neuropathy | 1 | - | - |

**eTable 2 Multivariate logistic regression of risk factors for irAEs in SCLC patients treated with immunotherapy.**

| **irAEs** | **Univariate Analysis** | | **Multivariate Analysis** | |
| --- | --- | --- | --- | --- |
|  | **OR (95%CI)** | ***P* value^a^** | **OR (95%CI)** | ***P* value^a^** |
| Age | 1.01（0.98-1.04） | 0.55 | 1.02（0.98-1.06） | 0.27 |
| Gender  (male vs female) | 0.84（0.44-1.61） | 0.60 | 0.52（0.20-1.36） | 0.18 |
| Smoking  (yes vs no) | 0.89（0.52-1.53） | 0.68 | 1.52（0.70-3.31） | 0.29 |
| ECOG PS  (0-1 vs ≥2) | 1.12（0.68-1.86） | 0.66 | 1.13（0.64-2.01） | 0.67 |
| Line of therapy for ICIs  (1st vs≥2nd) | 1.78（0.99-3.20） | 0.06 | 1.63（0.61-4.36） | 0.33 |
| Stage at diagnosis  (limited vs extensive) | 1.41（0.68-2.92） | 0.35 | 0.97（0.30-3.15） | 0.95 |
| Treament received  (PD-1 vs PD-L1) | 0.76（0.45-1.29） | 0.31 | 0.95（0.51-1.77） | 0.88 |
| Any history of brain metastases before ICI(yes vs no) | 1.42（0.81-2.47） | 0.22 | 1.59（0.84-3.00） | 0.16 |
| Any history of liver metastases before ICI (yes vs no) | 0.86（0.50-1.50） | 0.59 | 0.92（0.49-1.73） | 0.80 |
| ICI treatment cycles | 1.30（1.18-1.44） | **＜0.001** | 1.32（1.19-1.46） | **＜0.001** |

Abbreviations: ECOG PS, Eastern Cooperative Oncology Group performance status; ICI, immune checkpoint inhibitor; irAE, immune-related adverse event; PD-L1, programmed death-ligand 1; PD-1, programmed cell death protein 1.

^a^ Categorical and continuous variables were compared using χ2 and Kruskal-Wallis tests, respectively.

**eTable 3 Multivariate Cox regression model of overall and progression-free survival in SCLC patients treated with anti-PD(L)1 immunotherapy, utilizing irAEs as a time-varying covariate.**

|  | **PFS** | | | | **OS** | | | |
| --- | --- | --- | --- | --- | --- | --- | --- | --- |
|  | **Univariate Analysis** | | **Multivariate Analysis** | | **Univariate Analysis** | | **Multivariate Analysis** | |
|  | **HR (95%CI)** | ***P* value^a^** | **HR (95%CI)** | ***P* value^a^** | **HR (95%CI)** | ***P* value^a^** | **HR (95%CI)** | ***P* value^a^** |
| irAEs^b^  (yes vs no) | 0.94（0.91-0.97） | **＜0.001** | 0.93（0.90-0.97） | **＜0.001** | 0.99（0.96-1.02） | 0.67 | 0.99（0.97-1.03） | 0.80 |
| Age | 1.01（0.99-1.03） | 0.22 | 1.01（0.99-1.03） | 0.55 | 1.02（0.99-1.05） | 0.07 | 1.02（0.99-1.05） | 0.12 |
| Gender  (male vs female) | 1.47（1.02-2.12） | **＜0.05** | 1.27（0.80-2.03） | 0.32 | 1.41（0.81-2.47） | 0.22 | 1.03（0.51-2.10） | 0.93 |
| Smoking  (yes vs no) | 1.40（1.04-1.89） | **＜0.05** | 1.18（0.80-1.73） | 0.40 | 1.78（1.12-2.84） | **＜0.05** | 1.72（0.96-3.09） | 0.07 |
| ECOG PS  (0-1 vs ≥2) | 1.02（0.77-1.35） | 0.89 | 1.04（0.77-1.39） | 0.80 | 0.99（0.66-1.51） | 0.98 | 1.09（0.70-1.68） | 0.71 |
| Line of therapy for ICIs  (1st vs≥2nd) | 0.65（0.47-0.89） | **＜0.01** | 0.57（0.35-0.93） | **＜0.05** | 0.71（0.45-1.13） | 0.15 | 0.87（0.43-1.78） | 0.70 |
| Stage at diagnosis  (limited vs extensive) | 1.28（0.86-1.90） | 0.22 | 1.20（0.68-2.13） | 0.53 | 1.34（0.78-2.30） | 0.29 | 1.17（0.51-2.68） | 0.71 |
| Treatment received  (PD-1 vs PD-L1) | 1.28（0.96-1.71） | 0.09 | 1.21（0.88-1.67） | 0.23 | 1.58（1.04-2.41） | **＜0.05** | 1.68（1.07-2.64） | **＜0.05** |
| Any history of brain metastases before ICI(yes vs no) | 1.18（0.88-1.58） | 0.28 | 1.22（0.90-1.67） | 0.20 | 0.84（0.53-1.33） | 0.45 | 0.75（0.47-1.21） | 0.24 |
| Any history of liver metastases before ICI (yes vs no) | 1.35（1.01-1.82） | **＜0.05** | 1.37（1.01-1.87） | **＜0.05** | 1.23（0.79-1.93） | 0.36 | 1.12（0.70-1.79） | 0.63 |

Abbreviations: ECOG PS, Eastern Cooperative Oncology Group performance status; ICI, immune checkpoint inhibitor; irAE, immune-related adverse event; PD-L1, programmed death-ligand 1; PD-1, programmed cell death protein 1.

^a^ Categorical and continuous variables were compared using χ2 and Kruskal-Wallis tests, respectively.

^b^ irAEs are included in this model as time-varying covariate

**eTable 4 Multivariate Cox regression model of overall and progression-free survival** **in SCLC patients treated with anti-PD(L)1 immunotherapy, utilizing the three most common single irAEs as categorical variables.**

|  | **PFS** | | | | **OS** | | | |
| --- | --- | --- | --- | --- | --- | --- | --- | --- |
|  | **Univariate Analysis** | | **Multivariate Analysis** | | **Univariate Analysis** | | **Multivariate Analysis** | |
|  | **HR (95%CI)** | ***P* value^a^** | **HR (95%CI)** | ***P* value^a^** | **HR (95%CI)** | ***P* value^a^** | **HR (95%CI)** | ***P* value^a^** |
| Typle of irAE |  |  |  |  |  |  |  |  |
| No irAEs | Ref |  | Ref |  | Ref |  | Ref |  |
| Thyroid dysfunction | 0.40（0.29-0.53） | **＜0.001** | 0.37（0.27-0.50） | **＜0.001** | 0.62（0.40-0.96） | **＜0.05** | 0.65（0.42-1.02） | 0.06 |
| Rash | 0.30（0.16-0.56） | **＜0.001** | 0.27（0.14-0.51） | **＜0.001** | 0.72（0.30-1.70） | 0.45 | 0.63（0.26-1.54） | 0.31 |
| Pneumonitis | 0.38（0.19-0.73） | **＜0.01** | 0.36（0.18-0.72） | **＜0.01** | 0.41（0.13-1.32） | 0.13 | 0.44（0.13-1.45） | 0.18 |
| Age | 1.01（0.99-1.03） | 0.22 | 1.02（0.99-1.04） | 0.13 | 1.02（0.99-1.05） | 0.07 | 1.02（0.99-1.05） | 0.14 |
| Gender  (male vs female) | 1.47（1.02-2.12） | **＜0.05** | 1.11（0.70-1.76） | 0.67 | 1.41（0.81-2.47） | 0.22 | 0.96（0.47-1.94） | 0.90 |
| Smoking  (yes vs no) | 1.40（1.04-1.89） | **＜0.05** | 1.25（0.85-1.85） | 0.26 | 1.78（1.12-2.84） | **＜0.05** | 1.87（1.03-3.39） | **＜0.05** |
| ECOG PS  (0-1 vs ≥2) | 1.02（0.77-1.35） | 0.89 | 0.97（0.72-1.30） | 0.82 | 0.99（0.66-1.51） | 0.98 | 1.08（0.70-1.68） | 0.72 |
| Line of therapy for ICIs  (1st vs≥2nd) | 0.65（0.47-0.89） | **＜0.01** | 0.55（0.34-0.89） | **＜0.05** | 0.71（0.45-1.13） | 0.15 | 0.84（0.42-1.71） | 0.63 |
| Stage at diagnosis  (limited vs extensive) | 1.28（0.86-1.90） | 0.22 | 0.80（0.46-1.41） | 0.45 | 1.34（0.78-2.30） | 0.29 | 1.03（0.45-2.36） | 0.95 |
| Treatment received  (PD-1 vs PD-L1) | 1.28（0.96-1.71） | 0.09 | 1.23（0.90-1.68） | 0.20 | 1.58（1.04-2.41） | **＜0.05** | 1.84（1.17-2.87） | **＜0.01** |
| Any history of brain metastases before ICI(yes vs no) | 1.18（0.88-1.58） | 0.28 | 1.31（0.96-1.77） | 0.09 | 0.84（0.53-1.33） | 0.45 | 0.74（0.46-1.19） | 0.21 |
| Any history of liver metastases before ICI (yes vs no) | 1.35（1.01-1.82） | **＜0.05** | 1.32（0.97-1.80） | 0.08 | 1.23（0.79-1.93） | 0.36 | 1.02（0.63-1.64） | 0.95 |

Abbreviations: ECOG PS, Eastern Cooperative Oncology Group performance status; ICI, immune checkpoint inhibitor; irAE, immune-related adverse event; PD-L1, programmed death-ligand 1; PD-1, programmed cell death protein 1.

^a^ Categorical and continuous variables were compared using χ2 and Kruskal-Wallis tests, respectively.

**eTable 5 Multivariate Cox regression model of overall and progression-free survival in SCLC patients treated with anti-PD(L)1 immunotherapy, utilizing irAEs as a categorical variable.**

|  | **PFS** | | | | **OS** | | | |
| --- | --- | --- | --- | --- | --- | --- | --- | --- |
|  | **Univariate Analysis** | | **Multivariate Analysis** | | **Univariate Analysis** | | **Multivariate Analysis** | |
|  | **HR (95%CI)** | ***P* value^a^** | **HR (95%CI)** | ***P* value^a^** | **HR (95%CI)** | ***P* value^a^** | **HR (95%CI)** | ***P* value^a^** |
| Number of irAEs |  |  |  |  |  |  |  |  |
| No irAEs | Ref |  | Ref |  | Ref |  | Ref |  |
| Single-system irAEs | 0.43（0.32-0.58） | **＜0.001** | 0.41（0.30-0.55） | **＜0.001** | 0.73（0.48-1.12） | 0.15 | 0.74（0.48-1.15） | 0.18 |
| Multi-system irAEs | 0.30（0.17-0.52） | **＜0.001** | 0.28（0.15-0.51） | **＜0.001** | 0.31（0.11-0.86） | **＜0.05** | 0.35（0.12-0.99） | **＜0.05** |
| Age | 1.01（0.99-1.03） | 0.22 | 1.01（0.99-1.03） | 0.23 | 1.02（0.99-1.05） | 0.07 | 1.02（0.99-1.05） | 0.08 |
| Gender  (male vs female) | 1.47（1.02-2.12） | **＜0.05** | 1.18（0.73-1.90） | 0.50 | 1.41（0.81-2.47） | 0.22 | 1.06（0.51-2.19） | 0.88 |
| Smoking  (yes vs no) | 1.40（1.04-1.89） | **＜0.05** | 1.14（0.78-1.68） | 0.50 | 1.78（1.12-2.84） | **＜0.05** | 1.65（0.92-2.96） | 0.09 |
| ECOG PS  (0-1 vs ≥2) | 1.02（0.77-1.35） | 0.89 | 0.94（0.70-1.27） | 0.69 | 0.99（0.66-1.51） | 0.98 | 1.06（0.69-1.65） | 0.79 |
| Line of therapy for ICIs  (1st vs≥2nd) | 0.65（0.47-0.89） | **＜0.01** | 0.55（0.34-0.90） | **＜0.05** | 0.71（0.45-1.13） | 0.15 | 0.85（0.42-1.73） | 0.66 |
| Stage at diagnosis  (limited vs extensive) | 1.28（0.86-1.90） | 0.22 | 0.88（0.50-1.56） | 0.67 | 1.34（0.78-2.30） | 0.29 | 1.18（0.51-2.71） | 0.70 |
| Treatment received  (PD-1 vs PD-L1) | 1.28（0.96-1.71） | 0.09 | 1.14（0.83-1.57） | 0.43 | 1.58（1.04-2.41） | **＜0.05** | 1.60（1.01-2.52） | **＜0.05** |
| Any history of brain metastases before ICI(yes vs no) | 1.18（0.88-1.58） | 0.28 | 1.29（0.95-1.76） | 0.10 | 0.84（0.53-1.33） | 0.45 | 0.77（0.48-1.26） | 0.31 |
| Any history of liver metastases before ICI (yes vs no) | 1.35（1.01-1.82） | **＜0.05** | 1.30（0.95-1.78） | 0.11 | 1.23（0.79-1.93） | 0.36 | 1.06（0.66-1.71） | 0.80 |

Abbreviations: ECOG PS, Eastern Cooperative Oncology Group performance status; ICI, immune checkpoint inhibitor; irAE, immune-related adverse event; PD-L1, programmed death-ligand 1; PD-1, programmed cell death protein 1.

^a^ Categorical and continuous variables were compared using χ2 and Kruskal-Wallis tests, respectively.

**eTable 6 Multivariate Cox regression model of overall and progression-free survival in SCLC patients treated with anti-PD(L)1 immunotherapy, utilizing multi-system irAEs as a continuous variable.**

|  | **PFS** | | | | **OS** | | | |
| --- | --- | --- | --- | --- | --- | --- | --- | --- |
|  | **Univariate Analysis** | | **Multivariate Analysis** | | **Univariate Analysis** | | **Multivariate Analysis** | |
|  | **HR (95%CI)** | ***P* value^a^** | **HR (95%CI)** | ***P* value^a^** | **HR (95%CI)** | ***P* value^a^** | **HR (95%CI)** | ***P* value^a^** |
| Number of irAEs | 0.48（0.38-0.62） | **＜0.001** | 0.46（0.36-0.59） | **＜0.001** | 0.65（0.46-0.91） | **＜0.05** | 0.67（0.47-0.96） | **＜0.05** |
| Age | 1.01（0.99-1.03） | 0.22 | 1.01（0.99-1.03） | 0.18 | 1.02（0.99-1.05） | 0.07 | 1.02（0.99-1.05） | 0.10 |
| Gender  (male vs female) | 1.47（1.02-2.12） | **＜0.05** | 1.16（0.72-1.87） | 0.53 | 1.41（0.81-2.47） | 0.22 | 1.08（0.52-2.22） | 0.84 |
| Smoking  (yes vs no) | 1.40（1.04-1.89） | **＜0.05** | 1.13（0.77-1.66） | 0.53 | 1.78（1.12-2.84） | **＜0.05** | 1.66（0.93-2.98） | 0.09 |
| ECOG PS  (0-1 vs ≥2) | 1.02（0.77-1.35） | 0.89 | 0.93（0.69-1.25） | 0.63 | 0.99（0.66-1.51） | 0.98 | 1.07（0.69-1.66） | 0.76 |
| Line of therapy for ICIs  (1st vs≥2nd) | 0.65（0.47-0.89） | **＜0.01** | 0.55（0.34-0.89） | **＜0.05** | 0.71（0.45-1.13） | 0.15 | 0.85（0.42-1.73） | 0.66 |
| Stage at diagnosis  (limited vs extensive) | 1.28（0.86-1.90） | 0.22 | 0.91（0.51-1.60） | 0.72 | 1.34（0.78-2.30） | 0.29 | 1.17（0.51-2.69） | 0.71 |
| Treatment received  (PD-1 vs PD-L1) | 1.28（0.96-1.71） | 0.09 | 1.11（0.81-1.52） | 0.53 | 1.58（1.04-2.41） | **＜0.05** | 1.64（1.05-2.57） | **＜0.05** |
| Any history of brain metastases before ICI(yes vs no) | 1.18（0.88-1.58） | 0.28 | 1.27（0.93-1.73） | 0.13 | 0.84（0.53-1.33） | 0.45 | 0.78（0.48-1.26） | 0.31 |
| Any history of liver metastases before ICI (yes vs no) | 1.35（1.01-1.82） | **＜0.05** | 1.28（0.93-1.75） | 0.13 | 1.23（0.79-1.93） | 0.36 | 1.08（0.68-1.74） | 0.74 |

Abbreviations: ECOG PS, Eastern Cooperative Oncology Group performance status; ICI, immune checkpoint inhibitor; irAE, immune-related adverse event; PD-L1, programmed death-ligand 1; PD-1, programmed cell death protein 1.

^a^ Categorical and continuous variables were compared using χ2 and Kruskal-Wallis tests, respectively.

**eTable 7 Prognosis of patients with ≥ Grade 3 irAEs.**

| Observed indicators | Patients | | | | | | |
| --- | --- | --- | --- | --- | --- | --- | --- |
|  | 1 | 2 | 3 | 4 | 5 | 6 | All patients |
| Type of irAEs | liver disease | rash | mellitus | rash | pneumonitis | hyperthyroidism |  |
| Median PFS (Months) | 10.6 | 7.9 | 8.0 | 6.7 | 10.7 | 15.3 | 8.0(4.7-11.2)^a^ |
| Median OS (Months) | 25.3 | 16.2 | 16.6 | 12.5 | 13.7 | 17.5 | NA^b^ |

^a^ Median PFS in patients with ≥ Grade 3 irAEs.

^b^ Median OS in patients with ≥ Grade 3 irAEs.


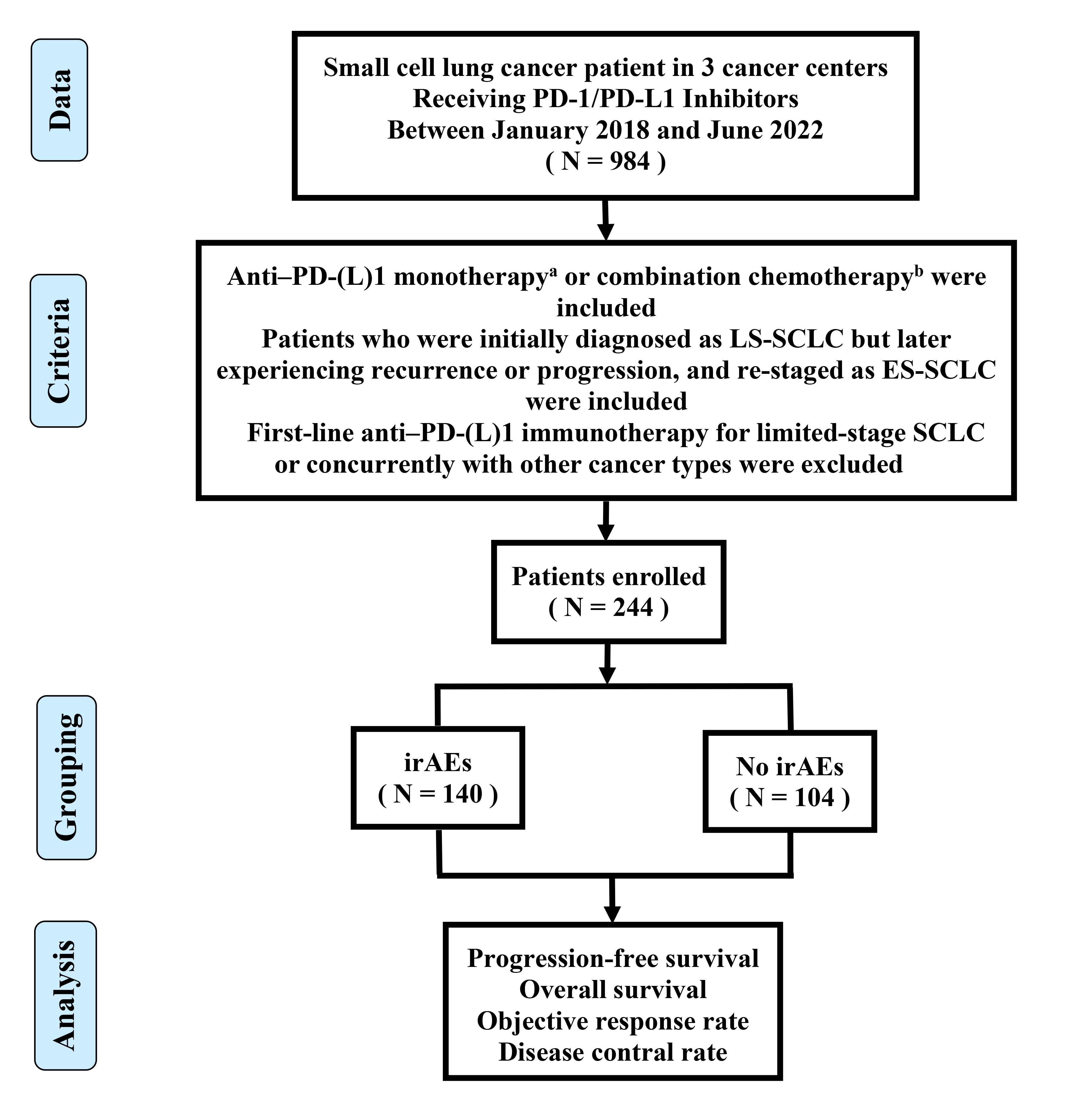


**eFigure 1. Study design: inclusion and exclusion criteria.**

**^a^**  Patients who received nivolumab and pembrolizumab monotherapy as third or later line for the treatment of patients with relapsed SCLC.

**^b^**  Patients who received etoposide and platinum as a first-line treatment and those treated with etoposide and platinum, irinotecan and platinum, or paclitaxel in second-line or beyond.


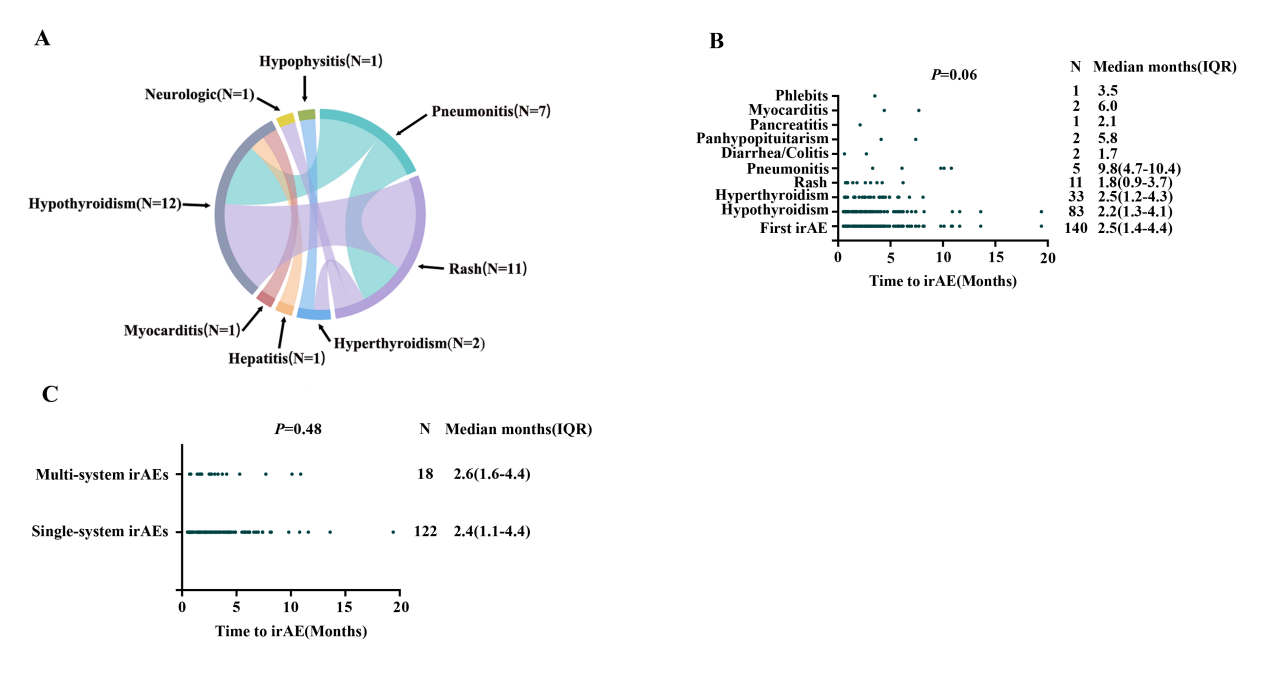


**eFigure 2. (A) Chord diagram displaying the spectrum of multi-system irAEs in SCLC patients treated with PD-1/PD-L1 inhibitors. (B and C) Time to onset of multigroup irAEs(B) or multi-system irAEs(C) in SCLC patients treated with PD-1/PD-L1 inhibitors.**
